# Plasma neurofilament light chain concentration is increased in anorexia nervosa

**DOI:** 10.1038/s41398-019-0518-2

**Published:** 2019-08-01

**Authors:** Ida A. K. Nilsson, Vincent Millischer, Virginija Danylaité Karrenbauer, Anders Juréus, Alireza M. Salehi, Claes Norring, Yvonne von Hausswolff-Juhlin, Martin Schalling, Kaj Blennow, Cynthia M. Bulik, Henrik Zetterberg, Mikael Landén

**Affiliations:** 1 Department of Molecular Medicine and Surgery, Karolinska Institutet, Stockholm, Sweden; 2 Center for Molecular Medicine, Karolinska University Hospital, Stockholm, Sweden; 3 Centre for Eating Disorders Innovation, Karolinska Institutet, Stockholm, Sweden; 4 Department of Clinical Neuroscience, Karolinska Institutet, Stockholm, Sweden; 5 Tema Neuro, PO3, Karolinska University Hospital, Stockholm, Sweden; 6 Department of Medical Epidemiology and Biostatistics, Karolinska Institutet, Stockholm, Sweden; 7 Centre for Psychiatry Research, Department of Clinical Neuroscience, Karolinska Institutet, Stockholm, Sweden; 8 Stockholm Health Care Services, Stockholm County Council, Stockholm, Sweden; 9 Stockholm Centre for Eating Disorders, Stockholm, Sweden; 10 Department of Psychiatry and Neurochemistry, Institute of Neuroscience and Physiology, The Sahlgrenska Academy at the University of Gothenburg, Mölndal, Sweden; 11 Clinical Neurochemistry Laboratory, Sahlgrenska University Hospital, Mölndal, Sweden; 12 Department of Psychiatry, University of North Carolina at Chapel Hill, Chapel Hill, NC USA; 13 Department of Nutrition, University of North Carolina at Chapel Hill, Chapel Hill, NC USA; 14 Department of Neurodegenerative Disease, UCL Queen Square Institute of Neurology, Queen Square, London, UK; 15 UK Dementia Research Institute at UCL, London, UK

**Keywords:** Molecular neuroscience, Psychiatric disorders

## Abstract

Anorexia nervosa (AN) is a severe psychiatric disorder with high mortality and, to a large extent, unknown pathophysiology. Structural brain differences, such as global or focal reductions in grey or white matter volumes, as well as enlargement of the sulci and the ventricles, have repeatedly been observed in individuals with AN. However, many of the documented aberrances normalize with weight recovery, even though some studies show enduring changes. To further explore whether AN is associated with neuronal damage, we analysed the levels of neurofilament light chain (NfL), a marker reflecting ongoing neuronal injury, in plasma samples from females with AN, females recovered from AN (AN-REC) and normal-weight age-matched female controls (CTRLS). We detected significantly increased plasma levels of NfL in AN vs CTRLS (median_AN_ = 15.6 pg/ml, IQR_AN_ = 12.1–21.3, median_CTRL_ = 9.3 pg/ml, IQR_CTRL_ = 6.4–12.9, and *p* < 0.0001), AN vs AN-REC (median_AN-REC_ = 11.1 pg/ml, IQR_AN-REC_ = 8.6–15.5, and *p* < 0.0001), and AN-REC vs CTRLS (*p* = 0.004). The plasma levels of NfL are negatively associated with BMI overall samples (*β* (±se) = −0.62 ± 0.087 and *p* = 6.9‧10^−12^). This indicates that AN is associated with neuronal damage that partially normalizes with weight recovery. Further studies are needed to determine which brain areas are affected, and potential long-term sequelae.

## Introduction

Anorexia nervosa (AN) is a psychiatric disorder characterized by persistent restriction of food intake resulting in significantly low body weight, combined with fear of gaining weight or behaviours that interfere with weight gain, and body image distortion^1,2^. The disorder affects ~1% of females and 0.1% of males, and an ~10% lethality makes it the most lethal psychiatric disorder^3–5^. On top of that, relapse rates and treatment failures are very common^6^. The pathoetiology has not been clarified, even though interactions among genetic, environmental, and neurobiological factors clearly contribute^1^. Twin studies have identified a strong genetic contribution, i.e., 58–70% of variance in liability is due to additive genetic factors^5,7,8^. Structural brain differences, most commonly global or focal reductions in grey matter or white matter (WM) volume, and sulci or ventricular enlargement, have repeatedly been shown in individuals with AN^9–11^. However, imaging studies of AN patients also exist that show no structural brain changes, particularly with regards to WM^12,13^. Importantly, structural changes reversed upon weight restoration in several studies^12,14–20^, while a few documented enduring changes^13,21,22^. This heterogeneity in results encourages to evaluate potential brain atrophy and neurodegeneration by complementary methods. Moreover, it is not known if the structural brain changes seen in AN are related to degeneration of brain cells or merely changes in fluids, as discussed by Ehrlich and et al.^23^. Combining results from well-designed imaging studies with analyses of markers in blood or cerebrospinal fluid (CSF) could help further explicate the neurobiology of AN.

In the present study, we hypothesize that neuronal injury and/or degeneration is involved in the pathophysiology of AN. To explore this, we measured the levels of neurofilament light chain (NfL), a marker reflecting ongoing neuronal damage, which can be reliably measured in serum, plasma or CSF^24–26^. We analysed plasma samples in a discovery cohort and a replication cohort, each stratified by three groups: (i) females with AN, (ii) females recovered from AN (AN-REC), and (iii) normal-weight age-matched female controls (CTRLS).

## Materials and methods

### Participants

For the discovery cohort, females with AN (*n* = 12) and weight-recovered females with a history of AN (AN-REC, *n* = 11) were recruited from Stockholm Centre for Eating Disorders (SCÄ). The general inclusion criteria for the AN group were female patients, at least 18 years old, meeting the DSM-IV criteria for AN^2^, and with at least 5 years since AN onset. AN-REC inclusion required weight recovery (BMI > 18) for at least 1 year. Normal-weight female controls (CTRLS, *n* = 12), without any own or family history of eating disorders were recruited via advertisements at Karolinska Institutet and internet (ki.se and studentkaninen.se).

For the replication cohort, participants were identified from the Swedish cohort of the Anorexia Nervosa Genetics Initiative (ANGI-SE), for details on the recruitment procedure see ref. ^27^. The general inclusion criteria for the AN replication group was female patients, at least 18 years old, meeting the DSM-IV criteria for AN^2^, with at least 1 year since AN onset (*n* = 112). For AN-REC, weight restoration (BMI > 20), no eating disorder behaviours for at least a year, and being within 1 SD of the mean for eating disorders cognitions (self-reports), were the inclusion criteria (*n* = 114). Age-matched normal-weight female controls had no history of disordered eating behaviour (CTRLS, *n* = 113). See Table 1 for further information on the study participants.

**Table 1:** Demographic and clinical characteristics of the study participants

Characteristics	AN	AN-REC	CTRL
Discovery cohort			
* n*	12	11	12
Females (%)	100	100	100
Age (years) (median [IQR])	31.0 (28.0–44.5)	28.0 (24.0–34.0)	27.5 (24.0–31.3)
BMI (kg/m^2^) (median [IQR])	14.8 (13.4–16.7)	19.8 (18.8–21.8)	24.1 (21.4–26.2)
Years since AN onset (median [IQR])	17.8 (10.5–27.3)	No info	–
Replication cohort			
* n*	112	114	113
Females (%)	100	100	100
Age (years) (median [IQR])	26.0 (24.0–31.0)	26.0 (24.0–31.0)	26.0 (24.0–31.0)
BMI (kg/m^2^) (median [IQR])	16.0 (15.0–17.0)	22.0 (21.0–24.3)	23.0 (22.0–26.0)
Years since AN onset (median [IQR])	10.0 (6.0–14.3)	10.0 (6.0–14.0)	–

*AN* anorexia nervosa, *AN-REC* recovered from anorexia nervosa, *CTRL* healthy controls, *IQR* interquartile range

The study was approved by the Regional Ethics Review Board in Stockholm. All participants provided oral and written informed consent to participate.

### Blood sampling and the NfL assay

For the discovery cohort venous blood was collected into vacutainer tubes including anticoagulant (sodium citrate) at SCÄ and processed within 2 h. For the replication cohort venous blood was collected into EDTA-tubes at the nearest hospital, mailed to Karolinska Institutet Biobank and processed upon arrival. After centrifugation, plasma samples were stored at −80 °C. Samples were transported on dry ice to the Clinical Neurochemistry laboratory at Sahlgrenska University Hospital where NfL concentration was measured using an in-house Single molecule array method as previously described in detail^28^. The measurements were performed in one round of experiments each for the discovery and replication cohorts. The intra- and inter-assay coefficients of variation were below 7% for QC samples with NfL concentrations of 20.4 pg/ml and 64 pg/ml, respectively.

### Statistical analyses

Demographic and clinical characteristics are presented using descriptive statistics.

Linear regression was used to analyse group differences (corrected for age) and to analyse the effects of age, BMI, and years since AN onset on plasma concentrations of NfL.

Statistical analyses were conducted using R programming language (including packages emmeans and multcomp ref. ^29^). Graphs were built using ggplot2 ref. ^30^. *P*-values <0.05 were considered statistically significant.

## Results

**Fig. 1:**
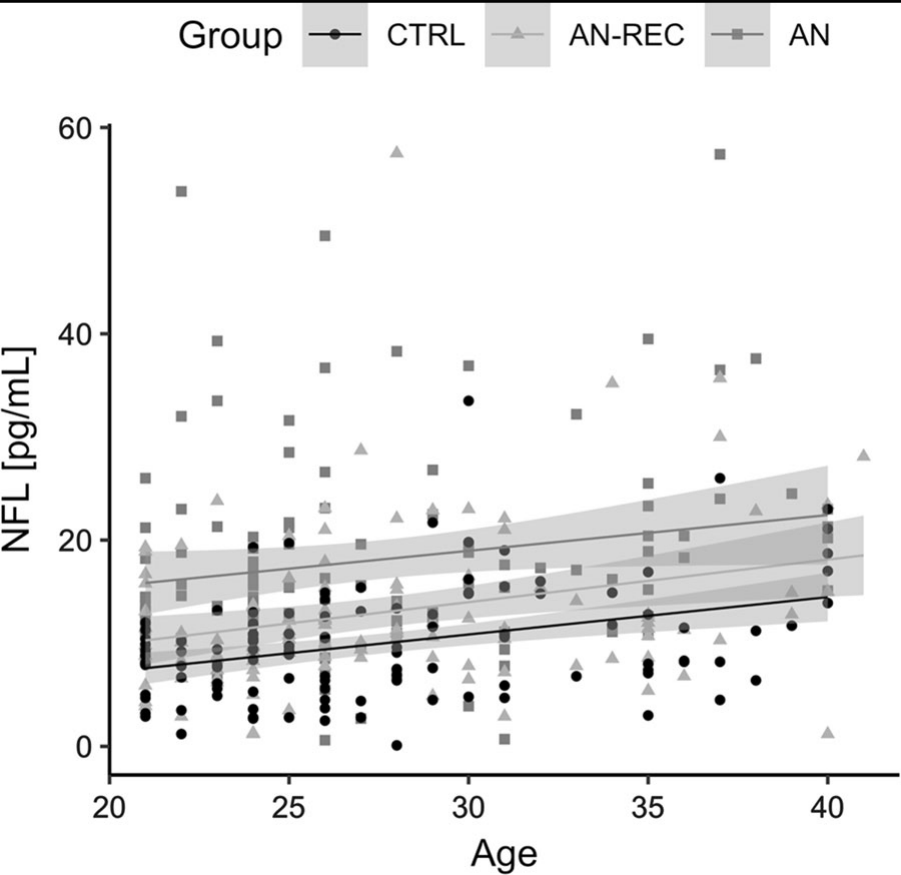
Plasma neurofilament light (NfL) levels are correlated with age in individuals with anorexia nervosa (AN), weight recovered from AN (AN-REC), and normal-weight healthy controls (CTRL). Overall age effect, *β* (±se) = 0.37 ± 0.087 and *p* = 2.7‧10^−5^. The shaded line around each linear fit line represents 95% confidence interval

Demographic and clinical characteristics of the study population are summarized in Table 1. As has been shown previously^31^, age at sampling was positively associated with plasma NfL levels, in both the discovery and the replication sample (Discovery sample: *β*_discovery_ (±se) = 0.45 ± 0.22 and *p* = 0.048; Replication sample: *β*_replication_ (±se) = 0.37 ± 0.087 and *p* = 2.7‧10^−5^) (Fig. 1). We therefore corrected for age in all subsequent statistical analyses.

**Fig. 2:**
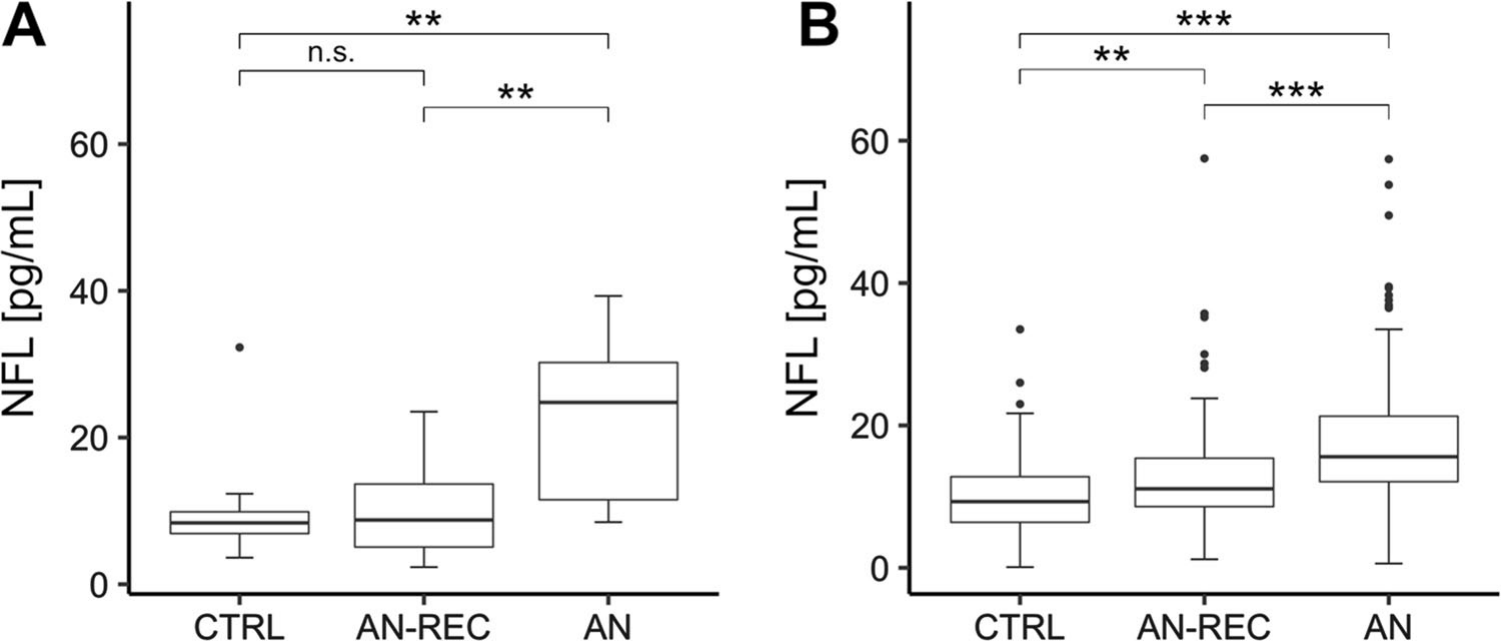
Box plot graphs showing plasma levels of neurofilament light (NfL) in individuals with anorexia nervosa (AN), weight recovered from AN (AN-REC) and normal-weight healthy controls (CTRL) in the discovery cohort (**a**) and the replication cohort (**b**). The median is shown as a straight line and the box denotes the interquartile range. *P*-values corrected for age, **p < 0.01 and ***p < 0.001

**Fig. 3:**
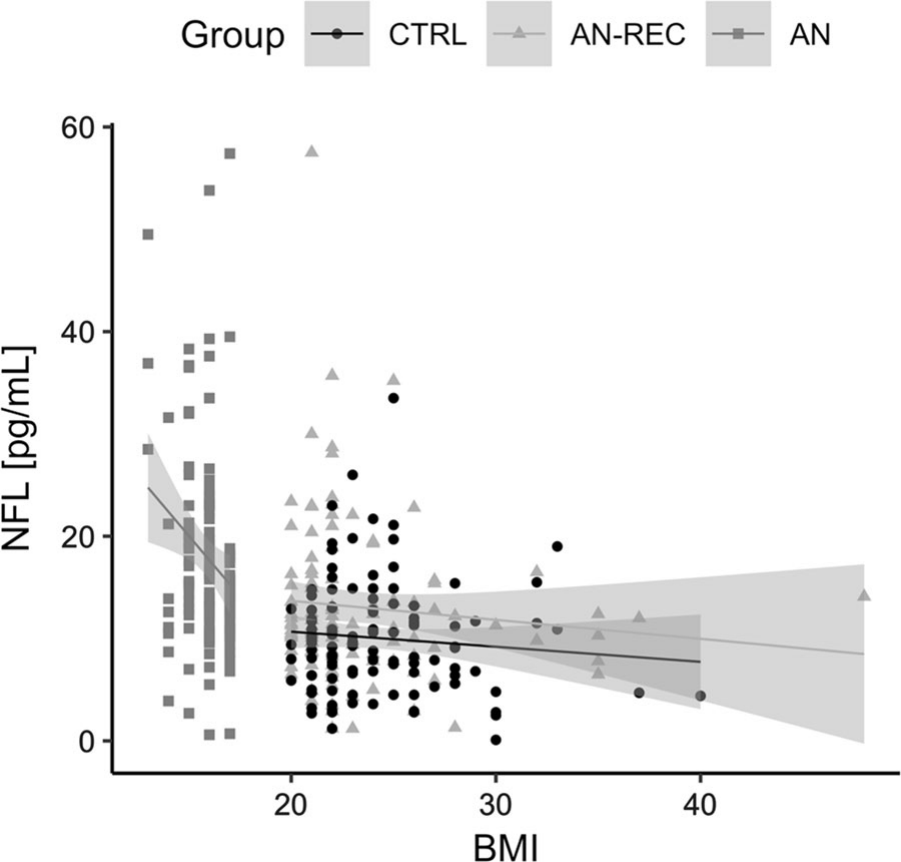
Plasma neurofilament light (NfL) levels are correlated with body mass index (BMI) in individuals with anorexia nervosa (AN), weight recovered from AN (AN-REC), and normal-weight healthy controls (CTRL). Overall BMI effect, *β* (±se) = −0.62 ± 0.087 and *p* = 6.9‧10^−12^. Note that the slope is significantly different in the AN group (−2.214) compared with AN-REC (−0.265 and *p* = 0.022) and CTRL (−0.190 and *p* = 0.018). The shaded line around each linear fit line represents 95% confidence interval

In both the discovery and replication sample, plasma levels of NfL were significantly higher in AN compared both with AN-REC (Discovery sample: median_AN_ = 24.8 pg/ml, IQR_AN_ = 10.8–30.7, median_AN-REC_ = 9.2 pg/ml, IQR_AN-REC_ = 5.1–15.9, and *p* = 0.005. Replication sample: median_AN_ = 15.6 pg/ml, IQR_AN_ = 12.1–21.3, median_AN-REC_ = 11.1 pg/ml, IQR_AN-REC_ = 8.6–15.5, and *p* = 1.64‧10^–6^) and with CTRLs (Discovery sample: median_CTRL_ = 7.8 pg/ml, IQR_CTRL_ = 4.7–9.9, and *p* = 0.005. Replication sample: median_CTRL_ = 9.3 pg/ml, IQR_CTRL_ = 6.4–12.9, and *p* = 1.16‧10^−13^). The levels in AN-REC were significantly higher than CTRLs only in the larger replication sample (*p*_discovery_ = 0.967 and *p*_replication_ = 0.004) (Fig. 2, see Table 2 for results of the linear model). Plasma NfL levels were negatively associated with BMI across all samples (*β* (±se) = −0.62 ± 0.087 and *p* = 6.9‧10^−12^). However, the slopes were significantly different in the AN group compared with the AN-REC and CTRL groups (*p* = 0.022 and 0.018, respectively). No significant difference in slopes was seen when comparing AN-REC with CTRL (*p* = 0.957) (Fig. 3).

**Table 2:** Results from linear regression analyses of plasma neurofilament light (NfL) levels and effects of group and age in the replication cohort

Independent variables	Estimate (β)	SE	*t*	*p*
Model 1 (adjusted *R*^2^ = 0.1906, ANOVA: *F* = 27.52, *n* = 339, *p* = 6.19 e−16)				
(intercept)	−0.356	2.340	−0.152	0.879
Group AN-REC	3.026	1.048	2.887	0.004
Group AN	8.153	1.053	7.743	1.16e−13
Age at sample	0.374	0.080	4.683	4.11e−06

*AN* anorexia nervosa, *AN-REC* recovered from anorexia nervosa

Finally, an association between the years since AN onset and NfL levels could be detected (*β* (±se) = 0.31 ± 0.11 and *p* = 0.006), but this association did not withstand correcting for age.

## Discussion

Here, we demonstrate significantly higher plasma levels of a marker for acute neuronal injury, NfL^24–26^, in female patients with AN, compared with normal-weight females with and without a history of AN. The difference in plasma NfL between AN-REC and CTRLS was smaller than the difference between AN and CTRLS suggesting that NfL might normalize somewhat with weight recovery. Elevated NfL levels have previously been documented in blood and/or CSF in several neurodegenerative conditions^32–36^, in ischaemic stroke^37^, and even in bipolar disorder^38^.

As to the origin of the neuronal injury proposed by the elevated NfL levels in AN, we can only speculate. Ehrlich et al. evaluated the levels of other blood markers related to neuronal or glial damage in AN: neuron-specific enolase, glial fibrillary acidic protein (GFAP) and S100B, in AN blood^23,39^. Contrary to our NfL findings, none of these three markers were altered in patients with active AN or recovered from AN, which might be explained by the substantially smaller number of study subjects in these former studies. Imaging studies of individuals with AN have, however, also yielded heterogeneous results. While several studies have documented structural grey matter or WM changes both globally and focally in AN, many but not all of these changes appear to reverse upon recovery^10^. Since NfL is predominantly increased upon axonal injury, WM is the primary suspect source of origin^26^. The list of areas in which structural WM reductions have been documented in AN includes, but is not limited to, the dorsal striatum^40^, the hippocampus^41^, the hippocampal-amygdala formation^42^, and the thalamus^43^. By diffusion tensor imaging and subsequent tractography analysis Florent and colleagues show reduced thickness of fibre tracts in a key food intake regulating hypothalamic area in AN compared with healthy normal weight and constitutionally lean controls (Florent, Baroncini, Jissendi-Tchofo, Lopes, Vanhoutte, Rasika, Pruvo, Vignau, Verdun, Johansen, Pigeyre, Bouret, Nilsson and Prevot, in preparation). The hypothalamus is an area of the brain that among others is crucial for the regulation of food intake and body weight, i.e., energy homeostasis^44^, an area in which signs of degeneration have been documented in the spontaneously anorectic *anx/anx* mouse^45^. Furthermore, reduced connectivity between the orbitofrontal cortex and amygdala to the hypothalamus^46^, as well as focal grey matter atrophy in the hypothalamus has been documented in AN^47^. It is tempting to speculate that degeneration of hypothalamic neurocircuitries responsible for energy homeostasis are involved in the paradoxical response to underweight seen in AN, i.e., the perpetual low food intake. In fact, AN genome wide association study have yielded significant negative single-nucleotide-based genetic correlations with BMI and other anthropometric measures^48–50^ which mechanistically could have its origin in the hypothalamus.

Another possibility is also that the elevated NfL levels in AN reflects a global reduction in brain volume as indicated by enlarged ventricles or CSF spaces/volumes and sulcal widening^10,12,18,21,51–55^, or even a peripheral neuropathy that might occur due malnutrition-related thiamine deficiency^56,57^.

The levels of NfL were negatively associated with BMI in AN, AN-REC, and CTRLS, but the slope was much steeper in the underweight BMI range/AN group. This is consistent with the findings that many of the structural brain differences reverse with recovery/normalization of body weight^10^, and have even been shown to correlate with weight loss^53^. Future studies should explore the levels of NfL in constitutionally lean individuals, in order to clarify if the increased plasma NfL in AN is exclusively an effect of low BMI.

One limitation of this study is that the levels of NfL were not compared with synchronized imaging in the same individuals. Another limitation is that since NfL levels reflect ongoing neuronal injury or degenerative process, we cannot determine the long-term and potentially permanent effects of the processes indicated by such increased levels. The difference in plasma NfL between AN-REC and CTRLS is smaller than the difference between AN and CTRLS, which could indicate that the levels normalize with time of recovery. The minimum recovery time was 1 year in the present study. It would be of value to evaluate the levels of NFL in plasma from individuals recovered from AN for a longer period, e.g., 5 years. But even if the levels would normalize completely with a longer time since recovery, it is still possible that the neuronal injury observed during active AN or short-term recovery has long-term structural and functional effects on the brain. Upregulation of brain derived neurotrophic factor has been documented in patients recovered from AN, which might indicate a regenerative response to a neuronal injury during the prolonged starvation^58^. Brain-imaging studies combined with long-term follow up evaluation of NfL levels are needed to clarify this.

Plasma and CSF levels of NfL are known to be very highly correlated^28^. It is however possible that the blood–brain barrier (BBB) becomes more permeable in starved conditions such as AN, which allows more NfL to leak into the circulation. In fact, in mice the BBB becomes more permeable after fasting^59^. Future studies should include evaluation of NfL in CSF of AN patients in order to explore a potential effect of a leaky BBB in AN.

To conclude, we report for the first time that plasma NfL levels are significantly increased in individuals with AN, compared with AN-REC and CTRLs. The levels were lower in recovered than underweight AN patients indicating that the active neuronal injury/degenerative process might attenuate upon recovery.
